# Association between obesity and age-related cataract: an updated systematic review and dose–response meta-analysis of prospective cohort studies

**DOI:** 10.3389/fnut.2023.1215212

**Published:** 2024-01-31

**Authors:** Sana Niazi, Majid Moshirfar, Mohammad H. Dastjerdi, Feizollah Niazi, Farideh Doroodgar, Renato Ambrósio

**Affiliations:** ^1^Translational Ophthalmology Research Center, Farabi Eye Hospital, Tehran, Iran; ^2^Ophthalmic Research Center, Research Institute for Ophthalmology and Vision Science, Shahid Beheshti University of Medical Sciences, Tehran, Iran; ^3^John A. Moran Eye Center, University of Utah, Salt Lake City, UT, United States; ^4^Department of Ophthalmology and Visual Science, Rutgers New Jersey Medical School, Newark, NJ, United States; ^5^Clinical Research Development Center, Shahid Modarres Educational Hospital, Shahid Beheshti University of Medical Sciences, Tehran, Iran; ^6^Negah Specialty Ophthalmic Research Center, Shahid Beheshti University of Medical Sciences, Tehran, Iran; ^7^Instituto de Olhos Renato Ambrósio, Rio de Janeiro, Brazil; ^8^Rio de Janeiro Corneal Tomography and Biomechanics Study Group, Rio de Janeiro, Brazil; ^9^BrAIN: Brazilian Artificial Intelligence Networking in Medicine, Rio de Janeiro, Brazil; ^10^Department of Ophthalmology, Federal University of the State of Rio de Janeiro (UNIRIO), Rio de Janeiro, Brazil; ^11^Department of Ophthalmology, Escola Paulista de Medicina, Universidade Federal de São Paulo, São Paulo, Brazil

**Keywords:** age-related cataract, body mass index, meta-analysis, obesity, cataract

## Abstract

**Objective:**

There are inconsistent findings on the association between obesity and age-related cataract (ARC). This systematic review was done to summarize available findings on the association between obesity [defined by body mass index (BMI)] and ARC by performing a dose–response meta-analysis on eligible prospective cohort studies.

**Methods:**

We performed a systematic search in PubMed, Scopus, ISI Web of Knowledge, and Google Scholar until June 2022 to identify eligible publications.

**Results:**

In total, 16 studies with a total sample size of 1,607,125 participants were included. Among all of these studies, there were 103,897 cases of ARC. In the follow-up periods ranging between 4 and 28 years, 4,870 cases of nuclear cataract, 1,611 cases of cortical cataract, and 1,603 cases of posterior subcapsular cataracts (PSC) were detected. By comparing the highest and lowest categories of BMI, we found that higher BMI was associated with an increased risk of ARC (RR: 1.18, 95% CI: 1.09–1.28) and PSC (RR: 1.44, 95% CI: 1.08–1.90). In the dose–response analysis, each 5 kg/m^2^ increase in BMI was associated with a 6 and 27% increased risk of ARC (RR: 1.06, 95% CI: 1.01–1.12) and PSC (RR: 1.27, 95% CI: 1.14–1.41), respectively. In addition, we found a positive association for cortical cataract among high-quality studies, in which higher BMI was associated with a 20% increased risk of cortical cataract (RR: 1.20, 95% CI: 1.02–1.42). In terms of nuclear cataract, we found no significant association either in the comparison between the highest and lowest categories of BMI or in the dose–response meta-analysis.

**Conclusion:**

Obesity (defined by BMI) was associated with an increased risk of ARC, PSC, and cortical cataract in adults. However, such a positive association was not seen for nuclear cataract.

**PROSPERO registration:**

CRD42022357132.

## 1 Introduction

Cataract is one of the top causes of visual impairment and blindness among the elderly (1, 2). The subtypes of cataracts include cortical, nuclear, and posterior subcapsular cataracts (PSC), with an age-standard pooled prevalence of 8.05, 8.22, and 2.24%, respectively, in the general population (3). In addition to morbidities, the presence of cataracts is associated with increased mortality (4).

Research has focused on age-related cataracts (ARC) as an inevitable aging issue, which has an increased risk in the presence of genetic and environmental factors (5). Among environmental and lifestyle factors, it has been shown that illiteracy, smoking, wine drinking, as well as underlying diseases such as hypertension and diabetes mellitus (DM) are the risk factors for ARC (6). Since obesity contributes to the etiology of DM and hypertension, it may affect the risk of ARC (7–9). However, the results from epidemiological studies investigating the association between obesity and ARC are inconsistent (10–25). In a cohort study of 1,312,051 adults, Floud et al. reported a significant positive association between obesity and ARC risk. However, some studies reported no significant association in this regard (10, 13, 14). Also, we found a cohort study in which obesity was associated with a reduced risk of ARC (22).

Two meta-analyses of Ye et al. (26) and Pan et al. (2), published in 2014, assessed the association between obesity and ARC and reported a significant positive association for ARC. However, findings from the two meta-analyses for cataract subtypes were different: Pan et al. reported a significant positive association in terms of nuclear and cortical cataracts, while Ye et al. did not find any significant association in this regard. It should be noted that these meta-analyses missed some eligible studies (14–18, 20, 25). In addition, these meta-analyses included some studies that were not eligible for this topic. For instance, in the association between body mass index (BMI) and ARC, they included the study of Chang et al. in which the link between weight change and ARC was evaluated (27). Also, Ye et al. (26) included the study of Lindblad et al. in which the relation between waist circumference and ARC was examined (28). These limitations may affect the results of these meta-analyses. Furthermore, none of these meta-analyses determined linear and non-linear dose–response associations between BMI and ARC. Therefore, the current systematic review and dose–response meta-analysis was conducted to determine the relation between BMI and ARC by summarizing available findings from prospective cohort studies.

## 2 Methods

### 2.1 Search strategy and study selection

This study was conducted using the preferred reporting items for systematic review and meta-analysis (PRISMA) standards (29). To find pertinent papers up to June 2022, we conducted a thorough search of the online databases of PubMed, Scopus, and ISI Web of Science. In the systematic search, we utilized both MeSH (medical subject heading terms) and non-MeSH terms (Supplementary Table 1). The publishing schedule and language of the pieces were both unrestricted. Following the thorough search, all of the results were imported into Endnote software before the screening process began. In Endnote, duplicate citations were eliminated. We also performed a web-based search in Google Scholar using the phrases “body mass index” and “cataract” in addition to the databases already listed. In order to ensure that we did not overlook any publications, we lastly checked the reference list of the chosen articles.

The following criteria were considered to select eligible studies in the screening step: (1) studies with prospective cohort designs, such as prospective cohort, nested case–control, and case-cohort studies; (2) studies on healthy adults (18 years); (3) studies measuring BMI to assess general obesity; (4) studies taking into account ARC or its subtypes, such as nuclear, cortical, or PSC as an outcome variable; and (5) studies reporting hazard ratio (HR), risk ratio (RR), and odds ratio (OR), with 95% confidence intervals for the association between obesity and ARC. If we found two papers that were published on a population, only the paper with higher quality or the most number of cases was included in our systematic review and meta-analysis. We disregarded retrospective, case–control, and cross-sectional studies as well as cohort studies that enrolled critically ill patients or people with chronic illnesses including diabetes mellitus and chronic kidney disease. Additionally, studies that did not report relative risks for the link between obesity and cataracts or lacked the necessary information to calculate these effect sizes were disregarded. Two independent reviewers chose the studies by taking the inclusion and exclusion criteria into account.

### 2.2 Data extraction and quality assessment

Two independent reviewers extracted data from each selected article and entered the data in an Excel-based form that was previously designed. Any discrepancies were discussed with a third reviewer in order to be rectified. Based on the form, the following information was extracted from each article: first author’s name, year of publication, cohort name, geographical region, characteristics of participants (age and gender), sample size, number of cases with ARC, follow-up period, methods used for the assessment of obesity and cataract, relative risk estimates, including ORs, RRs, and HRs for the link between obesity and ARC risk, and confounding variables adjusted in statistical analysis. If research did not offer the necessary estimations, we calculated them using conventional techniques.

We used the Newcastle Ottawa Scale (NOS), designed for prospective cohort studies, to assess the quality of included studies (30). Based on the NOS, each cohort study can get a maximum of nine points: four for the selection of participants, two for comparability, and three for the assessment of outcomes. In the current study, we categorized studies based on the median score of NOS in which studies with a score more than the median were considered high-quality ones.

### 2.3 Statistical analysis

We included the RRs, HRs, or ORs and 95% CIs reported for the association between obesity and ARC risk into the meta-analysis. These RRs were calculated based on the comparison between the highest versus lowest categories of BMI. However, it should be noted that some studies reported RRs of ARC per one-SD increment in BMI. To include in the meta-analysis, we converted the per SD increment risk estimates to the RRs for the comparison of the top versus bottom tertiles of BMI using the method suggested by Danesh et al. in which the log risk estimates reported for the comparison between the top and bottom tertiles of exposure variable are equivalent to 2.18 times of the log risk estimates for a 1-SD increase in that variable (31). This method assumes that the exposure is a normally distributed variable and that the association with disease risk is log-linear. To combine the RRs of ARC, a random-effects model was used. Random-effects models take into consideration different sources of uncertainties including within-study (sampling or estimation) error and between-studies variance (32, 33). To assess heterogeneity among studies, we used Cochran’s *Q* test and the *I*^2^ statistic. For the *I*^2^ statistic, we considered the I^2^ values of >50% as high between-study heterogeneity (34). To find possible sources of heterogeneity, subgroup analyses were conducted. Publication bias was examined using Egger’s linear regression test for the associations with more than 10 effect sizes (35). In the case of substantial publication bias, the trim-and-fill method was used to detect the effect of possibly missing studies on the overall RR (36). To assess the dependency of overall ES on one study, sensitivity analysis was done using a random-effects model in which each study was excluded to examine the influence of that study on the overall estimate.

Since the highest and lowest categories of BMI were different across the included studies, we performed a dose–response meta-analysis to determine the RR of ARC at different levels of BMI. We applied the method described by Crippa et al. to do a dose–response meta-analysis (37). In this method, the number of participants and cases of cataract and also the RR of ARC in each category of exposure (BMI) were required. In each category of BMI, we considered the median or mean amount of BMI as the corresponding RR of ARC. For studies that reported BMI as ranges, we estimated the midpoint in each category by calculating the mean of the lower and upper bound. When the highest or lowest category was open-ended, the length of the open-ended interval was assumed to be the same as that of the adjacent interval. We conducted the one-stage dose–response meta-analysis using restricted maximum likelihood estimation to assess linear and non-linear associations (37). This method estimates the study-specific slopes and combines them to obtain an overall average slope in a single stage, and is a more precise, flexible, and efficient method than the traditional two-stage method. Statistical analyses were conducted using STATA version 14.0. *p* < 0.05 was considered statistically significant for all tests, including Cochran’s *Q* test.

## 3 Results

### 3.1 Findings from the systematic search

In our initial search, we found 3,777 articles among the online databases, of them, 750 papers were duplicated. After excluding duplicate papers, we screened the remaining articles (*n* = 3,027) and disqualified any research that failed to fulfill the inclusion requirements (*n* = 2,997) (Supplementary Figure 1). After full-text reviews, nine articles were excluded because of being conducted on patients who underwent kidney transplantation or those with chronic diseases (*n* = 2) (38, 39), having a case–control or cross-sectional design (40–42), assessing waist circumference, weight or weight changes rather than BMI (28, 43, 44), and reporting incomplete data (45). Also, we excluded the study of Yuan et al. because they genetically predicted BMI (46). In addition, we found three different articles from the Physicians’ Health Study (21, 47, 48), two different papers from the UK Biobank (12, 49), and two different publications from the Blue Mountains Eye Study (22, 50). Since these publications evaluated similar associations, only the study with the highest quality or the greatest number of cases was considered for each dataset (12, 21, 22) and the duplicate papers were excluded (47–50). Moreover, two articles were published on the Beaver Dam Eye Study; however, both assessed different exposure and outcome variables in terms of BMI and cataract, and therefore, both were included (14, 16). After these exclusions, 16 articles containing 16 prospective cohort studies were included in the current systematic review and meta-analysis (10–25): 10 articles assessed BMI and risk of ARC (10–14, 21–25), 10 articles evaluated BMI and risk of nuclear cataract (13–15, 17–23), 6 papers assessed BMI and risk of cortical cataract (13, 16, 17, 20–22), and 7 publications assessed BMI and risk of PSC (13, 16, 17, 20–23). The flowchart of study selection is shown in Supplementary Figure 1.

### 3.2 Characteristics of studies

Characteristics of prospective studies included in the current systematic review and meta-analysis are shown in Table 1. The sample size of these studies ranged from 372 to 1,312,051 participants. In total, these studies recruited 1,607,125 participants with an age range of ≥40 years. In addition, during follow-up periods ranging between 4 and 28 years, 103,897 cases of ARC, 4870 cases of nuclear cataract, 1,611 cases of cortical cataract, and 1,603 cases of PSC were detected. The included articles were published between 1998 and 2016. Among the 16 articles, one article included only males (21), two articles performed analysis on only females (12, 19), and the remaining articles included both genders in statistical analysis (10, 11, 13, 15–18, 20, 22) or presented gender-stratified risk estimates (14, 23–25). In terms of geographical region, included studies were conducted in the US (11, 13, 14, 16, 18–21, 23, 24), Europe (10, 12, 15), Asia (17, 25), and Australia (22). In 7 articles, researchers measured weight and height using a standard protocol for calculating BMI (13, 15, 17–20, 22), while in 9 studies, self-reported weight and height were used (10–12, 14, 16, 21, 23–25). Regarding outcome assessment, researchers performed a direct examination for cataract diagnosis in four articles (13, 17–19) and used data from medical records/registries in 3 articles (10, 12, 15). Among the remaining articles, self-reported data were used for cataract assessment. Of the 16 included articles, 13 papers presented adjusted risk estimates for the association between BMI and cataract risk (10–16, 19, 21–25). Some important confounding variables including age (*n* = 12), smoking (*n* = 9), and having diabetes mellitus (*n* = 8) were adjusted in these studies. By considering the median NOS score of 7, 13 articles, of the 16 papers, had high quality or low risk of bias in most components of NOS (10–16, 19–24) (Supplementary Table 2).

**Table 1:** Characteristics of prospective cohort studies investigating the association between BMI and risk of ARC in adults.

Author	Country/cohort name	*n*	Age, y	Gender	Cases	follow-up	Exposure	Outcome	Outcome assess	Comparison (kg/m^2^)	ES	Adjustments
Floud et al. 2016	UK: UK biobank	1,312,051	50–64	Female	89,343	11	BMI: self-reported	Cataract surgery	Medical records/registries	BMI: ≥30 vs. <25	RR: 1.12 (1.10–1.14)	Age, residence, education, smoking, alcohol intake, physical activity, treatment for diabetes, age at menarche, parity, oral contraceptive use, hormone therapy
Kuang et al. 2013	Taiwan: SES	309	>65	Both	91	7	BMI: Measurement	Nuclear cataract	Examination	BMI: ≥25 vs. <25	RR: 1.04 (0.73–1.48)	None
		326			162			Cortical cataract		BMI: ≥25 vs. <25	RR: 0.92 (0.73–1.16)	
		372			30			PSC		BMI: ≥25 vs. <25	RR: 0.28 (0.10–0.79)	
Richter et al. 2012	US: LLES	3,187	>40	Both	196	4	BMI: Measurement	Nuclear cataract	Standard photographic grading	BMI: ≥30 vs. 18.5–25	RR: 0.89 (0.57–1.38)	None
		3,131			140			Cortical cataract		BMI: ≥30 vs. 18.5–25	RR: 1.10 (0.61–1.96)	
		3,007			16			PSC		BMI: ≥30 vs. 18.5–25	RR: 0.80 (0.17–3.73)	
Appleby et al. 2011	UK: EPIC-Oxford	27,670	>40	Both	1,484	11.4	BMI: self-reported	Total cataract	Medical records/registries	BMI: ≥27.5 vs. <20	IRR: 1.09 (0.92–1.29)	Age, sex, method of recruitment, residence, smoking
Karppi et al. 2011	Finland: KIHD	1,689	61–80	Both	108	4	BMI: Measurement	Nuclear cataract	Medical records/registries	BMI: T3 vs. T1 Per 1 SD increase	RR: 0.75 (0.45–1.21) RR: 0.97 (0.92–1.02)	Age, sex, smoking, alcohol consumption, serum LDL and HDL, education, corticosteroid use, history of diabetes and hypertension, current use of antihypertensive drugs
Mares et al. 2010	US: WHI	1808	50–79	Female	736	7	BMI: Measurement	Nuclear cataract	Examination	BMI: ≥35 vs. 22.5–25	OR: 1.61 (1.02–2.53)	Age, iris pigmentation, healthy Eating Index, smoking, pulse pressure, dietary variables, energy
Yoshida et al. 2010	Japan: JPHC	35,365	45–74	Male	1,004	5	BMI: self-reported	Total cataract	Self-reported	BMI: ≥25 vs. <19	OR: 1.15 (0.96–1.39)	Age, history of hypertension and diabetes, alcohol intake, smoking, PHC area
		40,825	45–74	Female	1807			Total cataract		BMI: ≥25 vs. <19	OR: 1.19 (1.04–1.36)	
Williams et al. 2009	US: NRHS	29,025	NR	Male	733	7	BMI: self-reported	Total cataract	Self-reported	BMI: ≥27.5 vs. <20 Per 1 unit increase	RR: 1.65 (1.04–2.84) RR: 1.03 (1.00–1.07)	Age, intake of meat, fish, fruit, and alcohol, physical activity
		11,967	NR	Female	179			Total cataract		Per 1 unit increase	RR: 0.97 (0.91–1.03)	
Tan et al. 2008	Australia: BMES	2,421	>48	Both	431	10	BMI: Measurement	Cataract surgery	Standard photographic grading	BMI: ≥30 vs. <25	RR: 0.67 (0.48–0.94)	Age, sex, sun-related skin damage, impaired fasting glucose, diabetes, steroids use, smoking, myopia, pulse pressure, diabetes, hypertension
		1782			498			Cortical cataract		BMI: ≥30 vs. <25	RR: 1.22 (0.91–1.64)	
		2013			182			PSC		BMI: ≥30 vs. <25	RR: 1.45 (0.92–2.28)	
		1,248			444			Nuclear cataract		BMI: ≥30 vs. <25	RR: 1.15 (0.82–1.61)	
Chodick et al. 2008	US: USRT	35,705	24–44	Both	2,315	20	BMI: self-reported	Total cataract	Self-reported	BMI: ≥30 vs. <20	HR: 1.44 (1.21–1.72)	Age, sex, marital status, education, iris color, skin complexion, hair color, ultraviolet exposure, smoking, alcohol intake, Hypercholesterolemia, Myocardial infarction, hypertension, arthritis, diabetes, intake of vitamin C, E, and multivitamin supplements, aspirin use
Leske et al. 2002	US, BES	2,609	40–84	Both	240	4	BMI: Measurement	Nuclear cataract	Examination	BMI: ≥25 vs. <25 Per 1 unit increase	RR: 0.64 (0.50–0.81) RR: 0.95(0.92–0.98)	None
Weintraub et al. 2002 (NHS)	US: NHS	49,259	>44	Female	3,241	16	BMI: self-reported	Total cataract	Self-reported	BMI: ≥30 vs. <23	RR: 1.39 (1.25–1.54)	Age, smoking, intake of Lutein/zeaxanthin
	US: HPFS	32,445		Male	1,189	10		Total cataract		BMI: ≥30 vs. <23	RR: 1.22 (0.97–1.54)	
	US: NHS	49,259		Female	993			Nuclear cataract		BMI: ≥30 vs. <23	RR: 1.02 (0.84–1.25)	
	US: HPFS	32,445		Male	268			Nuclear cataract		BMI: ≥30 vs. <23	RR: 1.21 (0.77–1.9)	
	US: NHS	49,259		Female	435			PSC		BMI: ≥30 vs. <23	RR: 2.05 (1.57–2.69)	
	US: HPFS	32,445		Male	138			PSC		BMI: ≥30 vs. <23	RR: 1.64 (0.86–3.15)	
Klein et al. 2003	US: BDES	2,710	43–84	Both	NR	5	BMI: self-reported	Cortical cataract	Standard photographic grading	BMI: T3 vs. T1 Per 1 unit increase	OR: 1.12 (0.89–1.59) OR: 1.01(0.99–1.04)	Age, sex
		2,863						PSC		BMI: T3 vs. T1 Per 1 unit increase	OR: 1.78 (1.26–2.76) OR: 1.05(1.02–1.09)	
Howard et al. 2014	Australia: BMES	1,131	43–84	Male	NR	15	BMI: self-reported	Cataract surgery	Standard photographic grading	BMI: T3 vs. T1	HR: 1.04 (0.78–1.43)	Age, physical activity, hypertension, diabetes
								Nuclear cataract		BMI: T3 vs. T1	HR: 0.74 (0.46–1.21)	
		1,480		Female	NR			Cataract surgery		BMI: T3 vs. T1	HR: 1.16 (0.91–1.46)	
								Nuclear cataract		BMI: T3 vs. T1	HR: 0.79 (0.58–1.07)	
Schaumberg et al. 2000	US: PHS	17,150	40–84	Male	1727	14	BMI: self-reported	Total cataract	Self-reported	BMI: ≥27.8 vs. <22	IRR: 1.2 (1–1.45)	Age, aspirin use, carotene intake, smoking, alcohol intake, diabetes mellitus, gout, systolic blood pressure, exercise, multivitamin use
		17,150			1,512			Nuclear cataract		BMI: ≥27.8 vs. <22	IRR: 1.26 (1.03–1.55)	
		17,150			652			Cortical cataract		BMI: ≥27.8 vs. <22	IRR: 1.18 (0.86–1.6)	
		17,150			721			PSC		BMI: ≥27.8 vs. <22	IRR: 1.38 (1.02–1.86)	
Hiller et al. 1998	US: FES	714	52–80	Both	444	28	BMI: Measurement	Total cataract	Examination	BMI: ≥27.8 vs. <22 Per 1 unit increase	OR: 1.38 (0.71–2.67) OR: 1.69 (0.8–3.55)	Age, sex, education, diabetes, smoking
		714			282			Nuclear cataract		BMI: ≥27.8 vs. <22 Per 1 unit increase	OR: 1.02 (0.52–2.02) OR: 1.04 (0.49–2.21)	
		714			159			Cortical cataract		BMI: ≥27.8 vs. <22 Per 1 unit increase	OR: 2.19 (0.98–4.92) OR: 1.91 (0.83–4.42)	
		714			81			PSC		BMI: ≥27.8 vs. <22 Per 1 unit increase	OR: 1.24 (0.45–3.42) OR: 6.13 (1.94–19.3)	

ARC, age-related cataract; BMI, body mass index; PSC, posterior subcapsular cataract; SES, Shihpai Eye Study; LLES, Los Angeles Latino Eye Study; KIHD, Kuopio Ischaemic Heart Disease Risk Factor Study; WHI, Women’s Health Initiative; PHS, Physicians’ Health Study; NRHS, National Runners’ Health Study; BMES, The Blue Mountains Eye Study (BMES); USRT, US Radiologic Technologists Study; BES, Barbados Eye Studies; BDES, Beaver Dam Eye Study; FES, Framingham eye study; COSM, Cohort of Swedish Men; NRHS, National Runners’ Health Study; SMC, The Swedish Mammography Cohort; NHS, Nurses’ Health Study; HPFS, Health Professionals Follow-up Study; JPHC, Japan Public Health Center-based Prospective Study.

### 3.3 Findings from the systematic review

Of the 10 articles that assessed the association between BMI and risk of ARC, 6 papers showed a significant positive association (11, 12, 21, 23–25) and others did not find any significant association. In addition, two articles, among the 10 publications on the link between BMI and risk of nuclear cataract, indicated a significant positive association (19, 21), while the remaining articles reported a non-significant association. None of the studies that examined the association between BMI and risk of cortical cataract revealed a significant association. In terms of BMI and risk of PSC, a significant positive association was reported in 3 articles (16, 21, 23) of the 7 publications.

### 3.4 Findings from the meta-analysis

In this section, we included all studies that were evaluated in the systematic review. Below, findings from the meta-analysis were reported for ARC and its subtypes.

#### 3.4.1 Meta-analysis on BMI and risk of ARC

**Figure 1:**
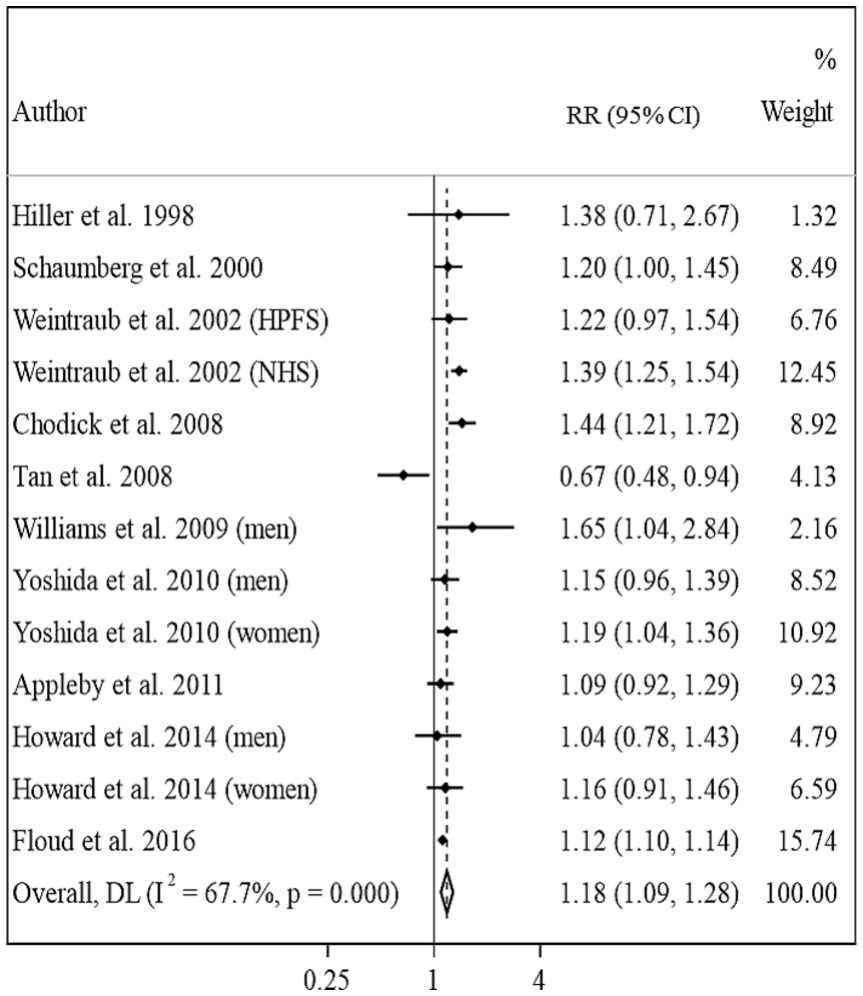
Forest plot for the association between BMI and ARC risk by comparing the highest and lowest categories of BMI. The overall RR was obtained from a random-effects model. RR, relative risk; CI, confidence interval; BMI, body mass index; ARC, age-related cataract; NHS, nurse health study; HPFS, health professional follow-up study.

Ten articles that included 11 studies assessed the association between BMI and risk of ARC (10–14, 21–25). The overall RR by comparing the highest and lowest categories of BMI was 1.18 (95% CI: 1.09–1.28, *I*^2^ = 67.7, *P*_heterogeneity_ < 0.001), indicating a significant positive association between BMI and ARC (Figure 1). However, between-study heterogeneity was significant in this regard. Subgroup analyses based on geographical region, gender, follow-up duration, and study quality reduced the heterogeneity, otherwise, these variables can be considered as possible sources of the observed heterogeneity. In the subgroup analyses, we found a significant positive association between BMI and risk of ARC in all subgroups except for studies that had a sample size of <10,000 participants.

**Figure 2:**
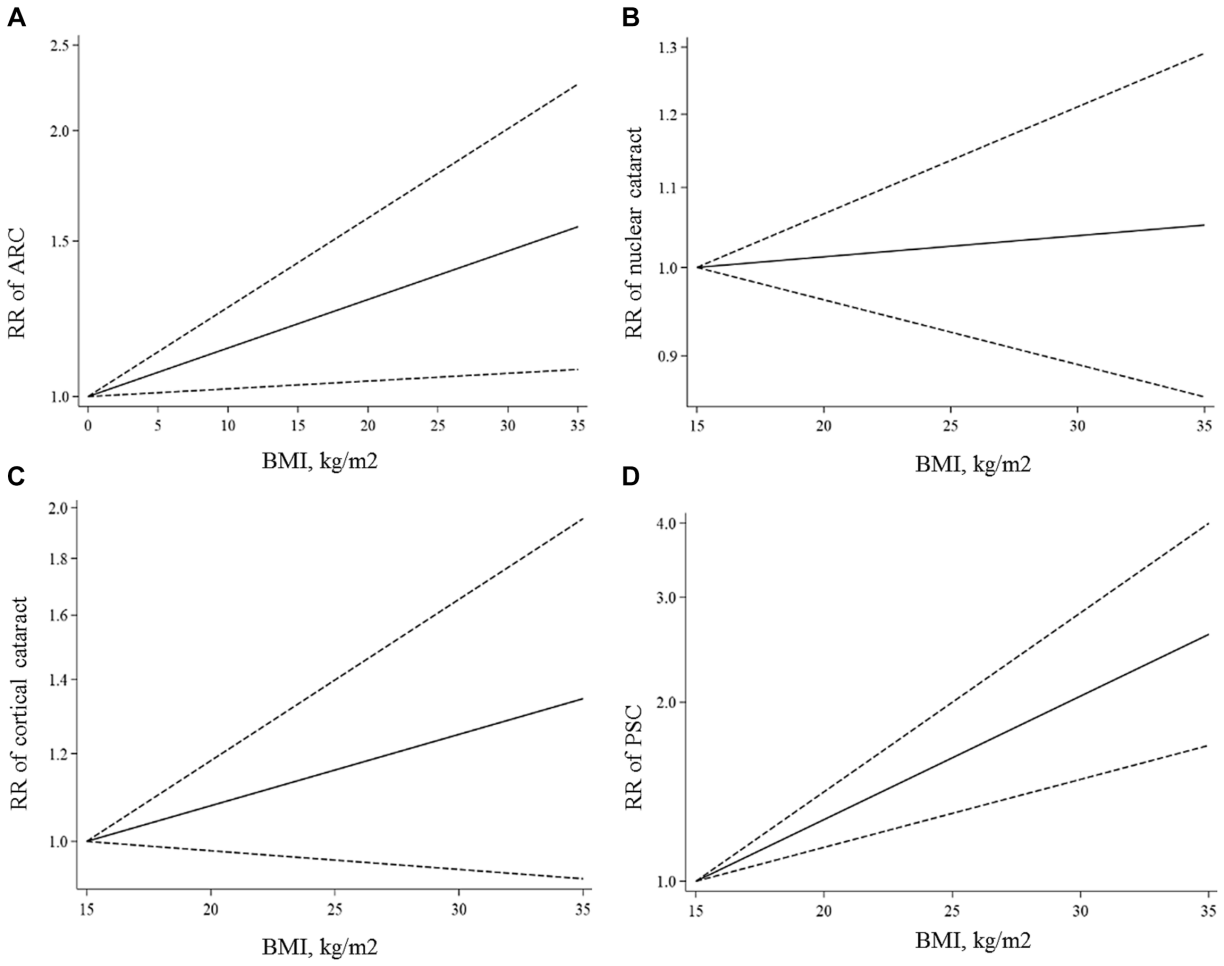
Linear dose–response associations of BMI with ARC **(A)**, nuclear **(B)**, cortical **(C)**, and PSC **(D)** cataracts. The solid lines indicate the overall RRs. The dashed lines present the 95% CIs. RR, relative risk; CI, confidence interval; BMI, body mass index; ARC, age-related cataract; PSC, posterior subcapsular cataract.

**Figure 3:**
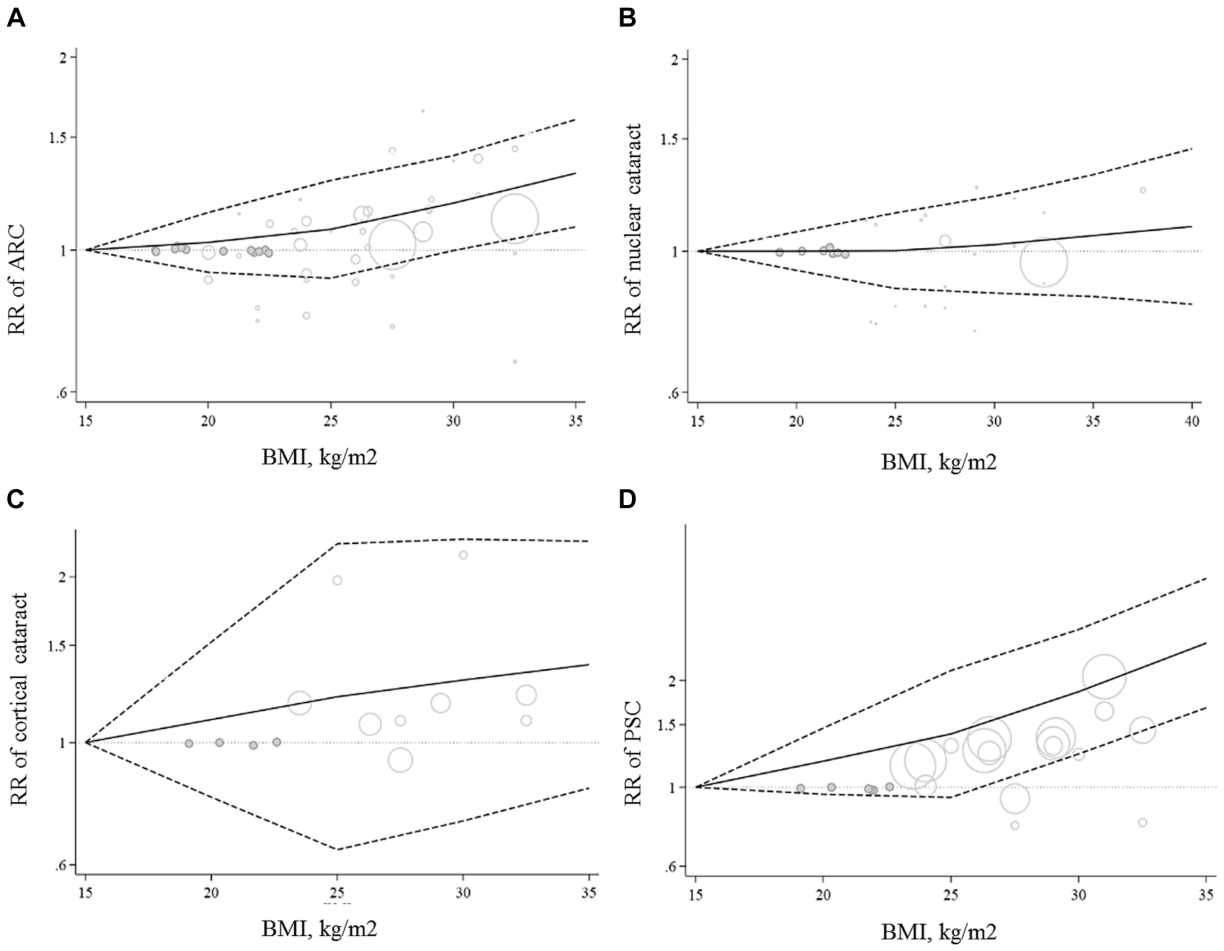
Non-linear dose–response associations of BMI with ARC **(A)**, nuclear **(B)**, cortical **(C)**, and PSC **(D)** cataracts. The solid lines indicate the overall RRs. The dashed lines present the 95% CIs. RR, relative risk; CI, confidence interval; BMI, body mass index; ARC, age-related cataract; PSC, posterior subcapsular cataract.

All articles in this section were included in the dose–response meta-analysis. We found a significant linear association between BMI and risk of ARC (Figure 2A); such that, the overall RRs of ARC per 1, 5, and 10 kg/m^2^ increase in BMI were 1.01 (95% CI: 1.00–1.02), 1.06 (95% CI: 1.01–1.12), 1.13 (95% CI: 1.02–1.26). In the non-linear dose–response meta-analysis, we found no evidence of a non-linear association between BMI and risk of ARC (P non-linearity = 0.39) (Figure 3A).

#### 3.4.2 Meta-analysis on BMI and risk of nuclear cataract

**Figure 4:**
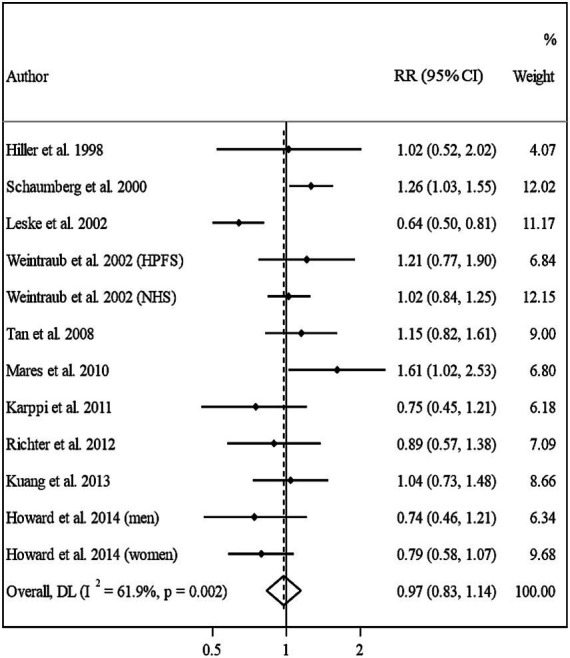
Forest plot for the association between BMI and the risk of nuclear cataract by comparing the highest and lowest categories of BMI. The overall RR was obtained from a random-effects model. RR, relative risk; CI, confidence interval; BMI, body mass index; ARC, age-related cataract.

Ten papers containing 11 studies were included in the meta-analysis of BMI and nuclear cataract (13–15, 17–23). Combining the RRs of nuclear cataract reported for the highest versus lowest categories of BMI revealed a non-significant association between BMI and nuclear cancer (Pooled RR: 0.97, 95% CI: 0.83–1.14, *I*^2^ = 61.9, *P*_heterogeneity_ = 0.002) (Figure 4). Such a non-significant association was also seen in the subgroup analyses (Table 2). In these analyses, we found that different characteristics of studies including geographical location, follow-up duration, sample size, and quality of included studies contributed to the significant heterogeneity observed in the overall analysis. In addition to the highest versus comparison, we found no significant association in the dose–response analysis (P linearity = 0.62, P non-linearity = 0.52) (Figures 2B, 3B).

**Table 2:** Subgroup analyses for the association between BMI and risk of ARC and its subtypes.

	#RR^1^	Pooled RR (95% CI)^2^	*P* ^3^	*I*^2^ (%)^4^	P-heterogeneity^5^
BMI and risk of total cataract					
Overall	13	1.18 (1.09–1.28)	<0.001	67.7	<0.001
Subgroup analysis					
Study location					
US	8	1.31 (1.21–1.41)	<0.001	8.3	0.36
Non-US countries	5	1.10 (1.00–1.21)	0.06	59.8	0.04
Gender					
Male	5	1.18 (1.07–1.31)	0.002	0	0.63
Female	4	1.21 (1.07–1.37)	0.002	82.0	0.001
Both	4	1.09 (0.80–1.49)	0.59	82.2	0.001
Follow-up, y					
≥10	10	1.17 (1.06–1.29)	0.002	73.8	<0.001
<10	3	1.19 (1.07–1.33)	0.001	0	0.41
Sample size, participants					
≥10,000	9	1.23 (1.13–1.33)	<0.001	70.5	0.001
<10,000	4	0.99 (0.75–1.32)	0.96	62.2	0.04
Adjustment for DM					
Adjusted	9	1.15 (1.05–1.26)	0.002	57.4	0.01
Non-adjusted	4	1.27 (1.09–1.48)	0.002	56.6	0.07
Study quality					
≥7	11	1.18 (1.07–1.31)	0.001	72.6	<0.001
<7	2	1.18 (1.06–1.31)	0.003	0	0.76
BMI and risk of nuclear cataract					
Overall	12	0.97 (0.83–1.14)	0.75	61.9	0.002
Subgroup analysis					
Study location					
US	9	0.97 (0.79–1.19)	0.77	70.1	0.001
Non-US countries	3	1.02 (0.82–1.27)	0.87	0	0.37
Gender					
Male	3	1.10 (0.81–1.48)	0.54	49.7	0.13
Female	3	1.05 (0.76–1.44)	0.77	69.4	0.04
Both	6	0.88 (0.70–1.10)	0.26	51.0	0.07
Follow-up, y					
≥10	7	1.04 (0.89–1.21)	0.62	34.2	0.16
<10	5	0.92 (0.66–1.27)	0.60	72.3	0.006
Sample size, participants					
≥10,000	3	1.14 (0.98–1.32)	0.08	8.7	0.33
<10,000	9	0.91 (0.74–1.10)	0.32	56.2	0.02
Adjustment for DM					
Adjusted	6	0.97 (0.78–1.20)	0.76	51.9	0.06
Non-adjusted	6	1.00 (0.77–1.28)	0.97	71.2	0.004
Study quality					
≥7	8	1.07 (0.92–1.26)	0.37	42.8	0.09
<7	4	0.80 (0.62–1.02)	0.07	45.1	0.14
BMI and risk of cortical cataract					
Overall	6	1.11 (0.96–1.28)	0.17	13.4	0.32
Subgroup analysis					
Study location					
US	4	1.18 (0.98–1.44)	0.08	0	0.48
Non-US countries	2	1.04 (0.79–1.37)	0.76	54.1	0.14
Follow-up, y					
≥10	3	1.25 (1.02–1.54)	0.03	0.7	0.36
<10	3	1.00 (0.84–1.19)	0.98	0	0.55
Sample size, participants					
≥10,000	1	1.18 (0.87–1.61)	0.29	–	–
<10,000	5	1.10 (0.92–1.33)	0.29	27.4	0.23
Adjustment for DM					
Adjusted	3	1.25 (1.02–1.54)	0.03	0.7	0.36
Non-adjusted	3	1.00 (0.84–1.19)	0.98	0	0.55
Study quality					
≥7	4	1.20 (1.02–1.42)	0.03	0	0.49
<7	2	0.94 (0.76–1.17)	0.59	0	0.57
BMI and risk of PSC					
Overall	8	1.44 (1.08–1.90)	0.01	57.9	0.02
Subgroup analysis					
Study location					
US	6	1.69 (1.43–2.01)	<0.001	1.3	0.40
Non-US countries	2	0.68 (0.14–3.40)	0.64	87.7	0.004
Gender					
Male	2	1.42 (1.08–1.87)	0.01	0	0.63
Female	1	2.05 (1.57–2.68)	<0.001	-	-
Both	5	1.10 (0.63–1.90)	0.73	64.7	0.02
Follow-up, y					
≥10	5	1.64 (1.36–1.99)	<0.001	10.6	0.34
<10	3	0.78 (0.21–2.83)	0.70	82.2	0.004
Sample size, participants					
≥10,000	3	1.69 (1.27–2.24)	<0.001	46.2	0.15
<10,000	5	1.10 (0.63–1.90)	0.73	64.7	0.02
Adjustment for DM					
Adjusted	3	1.39 (1.09–1.77)	0.008	0	0.95
Non-adjusted	5	1.33 (0.81–2.19)	0.25	72.3	0.006
Study quality					
≥7	6	1.67 (1.43–1.96)	<0.001	0	0.46
<7	2	0.40 (0.15–1.06)	0.06	17.8	0.27

ARC, age-related cataract; BMI, body mass index; PSC, posterior subcapsular cataract; US, United States; DM, diabetes mellitus.

^1^Number of effect sizes.

^2^Obtained from the random-effects model.

^3^Referred to the 95% CIs.

^4^Inconsistency- the percentage of variation across studies due to heterogeneity.

^5^Obtained from the *Q*-test.

#### 3.4.3 Meta-analysis on BMI and risk of cortical cataract

**Figure 5:**
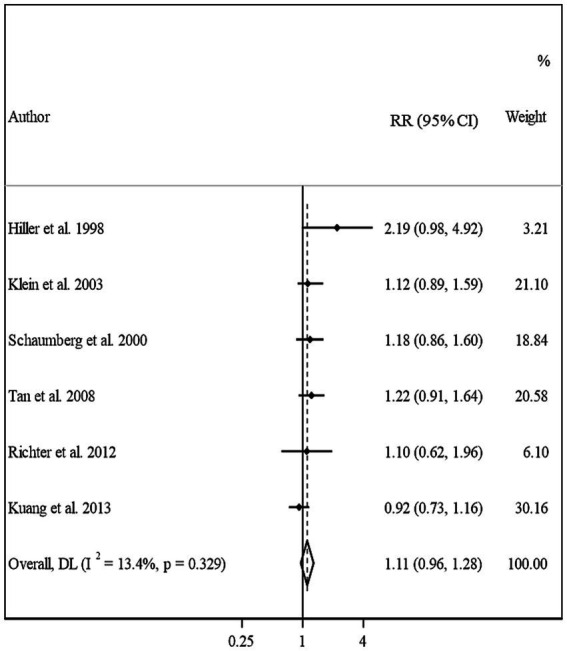
Forest plot for the association between BMI and the risk of cortical cataract by comparing the highest and lowest categories of BMI. The overall RR was obtained from a random-effects model. RR, relative risk; CI, confidence interval; BMI, body mass index; ARC, age-related cataract.

In total, we included 6 studies (from 6 papers) in this meta-analysis (13, 16, 17, 20–22). There was no significant association between BMI and risk of cortical cataract when we compared risk between the highest and lowest categories of BMI (Pooled RR: 1.11, 95% CI: 0.96–1.28, *I*^2^ = 13.4, *P*_heterogeneity_ = 0.32) (Figure 5). Between-study heterogeneity was not significant in this association. Regarding subgroup analyses, we found a significant positive association between BMI and risk of cortical cataract among cohort studies with a follow-up duration of ≥10 years (Pooled RR: 1.25, 95% CI: 1.02–1.54, *I*^2^ = 0.7, P_heterogeneity_ = 0.36), those that adjusted for diabetes in their analysis, and those with high quality (Pooled RR: 1.20, 95% CI: 1.02–1.42, *I*^2^ = 0, P_heterogeneity_ = 0.49) (Table 2). In the dose–response analysis of four articles containing required data, we found no evidence of linear (P linearity = 0.12) or non-linear (P non-linearity = 0.90) association between BMI and risk of cortical cataract (Figures 2C, 3C).

#### 3.4.4 Meta-analysis on BMI and risk of PSC

Overall, eight studies from seven articles were assessed in the meta-analysis on BMI and risk of PSC (13, 16, 17, 20–23). We found a significant positive association in this regard; such that, people in the highest categories of BMI had a 44% higher risk of PSC compared with those in the lowest category (Pooled RR: 1.44, 95% CI: 1.08–1.90, *I*^2^ = 57.9, *P*_heterogeneity_ = 0.02) (Figure 5). However, we found evidence of moderate heterogeneity in this association. Subgroup analyses showed that participants’ gender, follow-up duration, study location, and study quality were possible reasons for the observed heterogeneity (Table 2). From these analyses, we also found a significant positive association between BMI and PSC risk among cohort studies conducted in the US and those with high quality such as studies with high follow-up duration, those with larger sample sizes, and studies that controlled their analysis for diabetes.

In the dose–response meta-analysis, five papers (6 studies) on the link between BMI and PSC had required data, and therefore, were included in the dose–response meta-analysis (13, 20–23). There was evidence of a linear association between BMI and risk of PSC (Figure 2D) so that each 1, 5, and 10 kg/m2 increase in BMI was associated with a 5% (Pooled RR: 1.05, 95% CI: 1.03–1.07), 27% (Pooled RR: 1.27, 95% CI: 1.14–1.41), and 61% (Pooled RR: 1.61, 95% CI: 1.30–2.00) higher risk of PSC in adults. We found no evidence of a non-linear association in this regard (Figure 3D).

#### 3.4.5 Publication bias and sensitivity analysis

**Figure 6:**
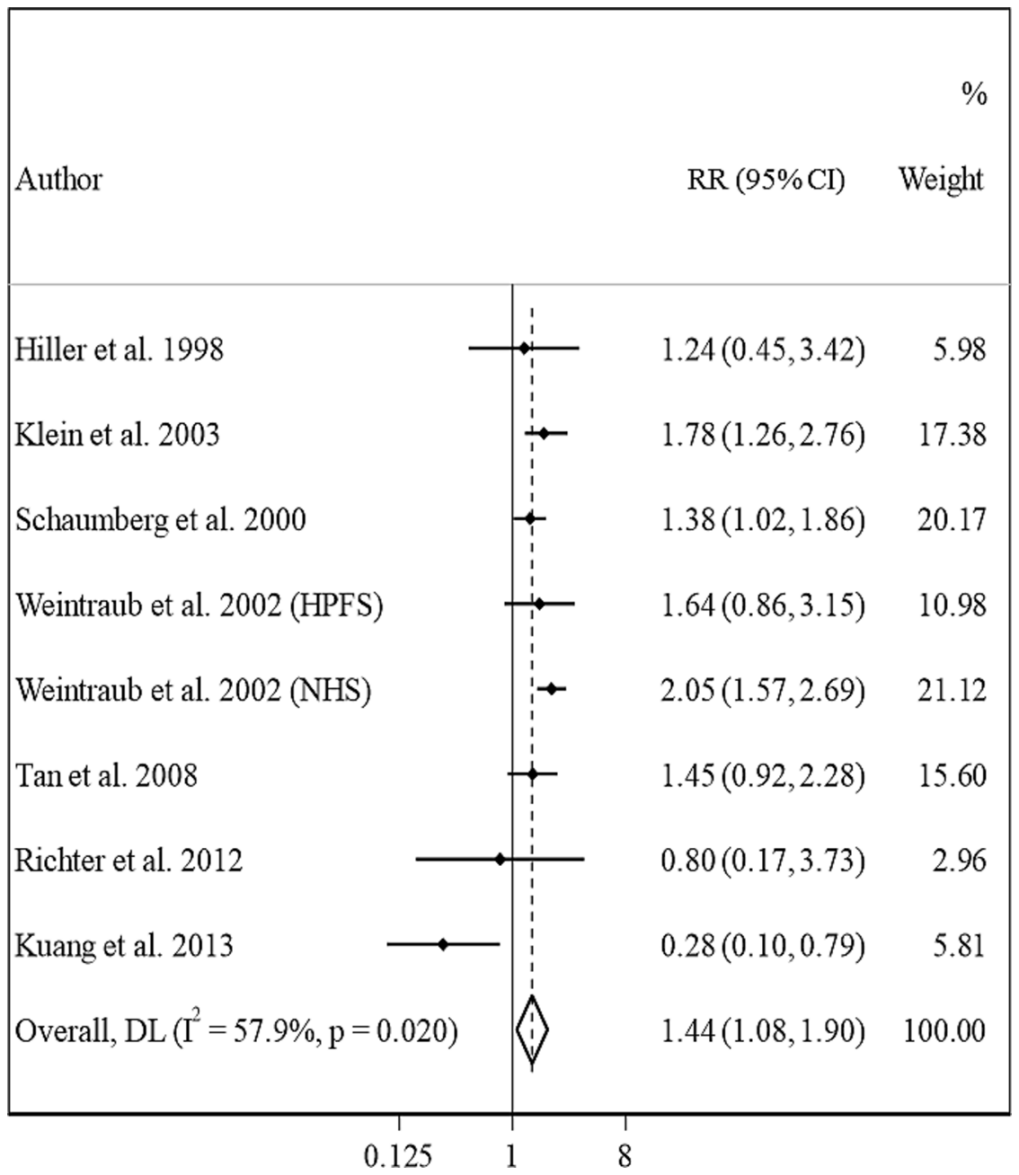
Forest plot for the association between BMI and the risk of PSC by comparing the highest and lowest categories of BMI. The overall RR was obtained from a random-effects model. RR, relative risk; CI, confidence interval; BMI, body mass index; ARC, age-related cataract; PSC, posterior subcapsular cataract.

In the sensitivity analysis, when we excluded the study of Leske et al., the non-significant positive association between BMI and risk of nuclear cataract became significant (Pooled RR: 1.11, 95% CI: 1.01–1.22). Sensitivity analyses for other associations showed that the overall RRs obtained in the current meta-analysis were robust and did not depend on one study. We assessed publication bias using Egger’s linear regression test for associations with ≥10 risk estimates and found no substantial publication bias (Figure 6).

## 4 Discussion

In the current meta-analysis, we found a significant positive association between BMI and risk of ARC and PSC in adults so that each 5 kg/m2 increase in BMI was associated with a 6 and 27% increased risk of ARC and PSC, respectively. In terms of nuclear and cortical cataracts, we found no significant association in the overall analysis; however, in the subgroup analyses, a significant positive association was seen between BMI and cortical cataract among studies with high quality.

ARC is a common disorder among older adults (51). Previous studies have shown that lifestyle-related factors such as smoking, alcohol consumption, and exposure to radiation or environmental pollution contribute to the etiology of ARC (52–54). However, the genetic potential of individuals plays an important role (55). Recently, some cohort studies have shown that obesity may affect the risk of cataract among older adults (10–25). However, findings from these studies were not conclusive. In the current meta-analysis, we found that higher BMI was associated with an increased risk of ARC. Also, in the dose–response meta-analysis, a 5 kg/m^2^ increase in BMI was associated with a 6% higher risk of ARC. In a 2014 meta-analysis, Ye et al. reported that obesity is a potential risk factor for ARC (26). In another review article, Line et al. concluded that obesity has a direct association with age-related eye diseases (56). Despite the positive association, some cohort studies included in the current meta-analysis indicated a non-significant association between BMI and risk of ARC (10, 13, 14, 22). This might be explained by the different sample sizes of the studies that were mostly low. Also, in the subgroup analyses, we found a significant positive association between BMI and ARC among studies with higher sample sizes, while this was not significant among small cohorts. In addition, different adjustments in the statistical analysis might be another reason for inconsistent results among the included studies. For instance, three studies that did not include any confounders in models showed no significant association between BMI and ARC and surprisingly indicated a significant inverse association for nuclear and PSC cataracts (17, 18, 20). In contrast, most studies that controlled their analysis for potential confounders revealed a significant positive association between BMI and ARC risk. Further studies are needed to confirm the positive association.

In the current study, we found a significant positive association between BMI and the risk of PSC. Also, each 5 kg/m^2^ increase in BMI was associated with a 27% higher risk of PSC. This was in line with a previous meta-analysis in which elevated BMI increased the risk of PSC. However, the dose–response association between BMI and PSC was not assessed in that meta-analysis (26). Also, in a prospective cohort study, Lindblad et al. reported that metabolic syndrome with the combination of abdominal adiposity, diabetes, and hypertension was associated with an increased risk for cataract extraction (28). Such a positive association was also reported in another cohort study (49). Among 7 papers included in the meta-analysis of BMI and PSC (13, 16, 17, 20–23), 3 articles reported a significant positive association (16, 21, 23), 3 indicated no significant association (13, 20, 22), and one showed an inverse association between BMI and PSC risk (17). The lack of significant positive association among the four studies might be due to the short duration of follow-up, low sample size, and totally low quality of these studies. The involvement of these variables was confirmed in the subgroup analyses in which we found a significant positive association between BMI and PSC risk in studies with high quality, long duration of follow-up (≥10 years), and high sample size (≥10,000 participants).

Elevated BMI or obesity is associated with several complications such as diabetes mellitus, hypertension, and hyperlipidemia (57, 58). These complications are known risk factors of ARC (59). In the subgroup analyses, we found a significant positive association between BMI and ARC among studies that adjusted for diabetes mellitus in their analysis. It means that there are other plausible pathophysiological pathways in addition to obesity complications through which elevated BMI increases the risk of ARC. It has been shown that obese individuals have increased levels of leptin which has a role in the elevation of oxidative stress (60). The role of oxidative stress in the progression of ARC has been well-established (61). In addition, obesity is linked with increased levels of inflammatory biomarkers which are involved in the development of ARC (62, 63).

In the current study, BMI had no significant association with nuclear and cortical cataracts in the overall analysis, however, in the subgroup analyses, a significant positive association was seen between BMI and cortical cataract among studies with high quality. In contrast with our findings, a 2014 meta-analysis showed a significant positive association between obesity and risk of nuclear and cortical cataracts (64). This inconsistency is explained by entering eligible articles, published after 2014, into the current meta-analysis. Unlike the cortical cataract, we found no significant association between BMI and nuclear cataract in any subgroups of the included studies. The lack of significant association for nuclear cataract might be due to the different patterns of formation and progression of this subtype compared with other subtypes of cataracts (65). For instance, PSC is highly overrepresented among extracted cataracts, while other subtypes are less common (61). Therefore, this might be a reason for the stronger association between BMI and PSC compared with other subtypes of cataracts.

Strengths of our meta-analysis included the linear and non-linear dose–response analyses on prospective cohort studies, which help us to draw the shape of the association between BMI and ARC. Since we included prospective cohort studies in the current meta-analysis, our findings are less susceptible to recall and selection bias which is common among retrospective case–control studies. In addition, to combine RRs, we used a random-effects model, that takes between-study variation into account. Despite the strengths, our meta-analysis had some limitations that should be considered when interpreting our results. The methods used for the definition of cataract were different among the included studies and some defined cataract based on self-reported data that may induce underestimates of the number of cataract cases. This problem was also the case for BMI which was calculated based on self-reported weight and height in some studies. Furthermore, in the comparison between the highest and lowest categories of BMI, we observed different cut-off points for the definition of these categories among included studies. However, we handled this problem by performing the dose–response meta-analysis.

In the current meta-analysis, we concluded that increased BMI is associated with a higher risk of ARC, particularly PSC, in adults. Moreover, we found that a 5 kg/m2 increase in BMI was associated with a 6 and 27% increased risk of ARC and PSC, respectively. We also found a significant positive association between BMI and risk of cortical cataract in high-quality studies. No significant association was seen for nuclear cataract. Future studies should assess the link between abdominal obesity and the risk of ARC.

## Author contributions

SN and MM contributed to the literature search and data extraction. MD and SN contributed to data analysis. FD and FN drafted the manuscript which was critically revised for important intellectual content by all authors. RA contributed to the manuscript editing. FD supervised the study. All authors have read and approved the final manuscript.
