# Supplementary material for: Association between obesity and age-related cataract: an updated systematic review and dose–response meta-analysis of prospective cohort studies
Source: Front Nutr. 2024 Jan 31;10:1215212. doi: 10.3389/fnut.2023.1215212 (PMC10866009; doi:10.3389/fnut.2023.1215212)
Supplement: Supplementary file 1 [file Data_Sheet_1.docx]

**Supplementary Table 1**: The terms used to search relevant publications on the relation between BMI and ARC

| **In PubMed, Scopus, and ISI Web of Science** | | **n** |
| --- | --- | --- |
|  | 1. “body mass index” |  |
|  | 2. "BMI" |  |
|  | 3. “obesity” |  |
|  | 4. “overweight” |  |
|  | 5. “weight” |  |
|  | 6. (1 OR 2 OR 3 OR 4 OR 5) |  |
|  | 7. cataract |  |
|  | 8. “lens opacity” |  |
|  | 9. (16 OR 17) |  |
|  | (6 AND 9) **In PubMed** | 1371 |
|  | (1 AND 9) **In Scopus** | 747 |
|  | (1 AND 9) **In ISI Web of Science** | 1159 |
|  | **Duplicate** | 750 |
| **In Google Scholar** | | 500 |
|  | “body mass index” and cataract  By searching the above combination in this engine, we screened the first 500 relevancy ranked papers to avoid missing any eligible studies. |  |
| **Total** | | 3027 |

Abbreviations: BMI: body mass index, ARC: age-related cataract

**Supplementary Table 2**: Quality assessment of prospective cohort studies investigating the association between BMI and ARC ^1^

|  | Representativeness of the exposed cohort | Selection of the non-exposed cohort | Ascertainment of exposure | Outcome of interest was not present at the start of the study | Age adjustment | Controls for any additional factor | Assessment of outcome | Follow-up long enough | Adequacy of follow-up of cohorts | Total |
| --- | --- | --- | --- | --- | --- | --- | --- | --- | --- | --- |
| Appleby et al. 2011 | * | * |  | * | * | * | * | * | * | 8 |
| Chodick et al. 2008 | * | * |  | * | * | * |  | * | * | 7 |
| Floud et al. 2016 | * | * |  | * | * | * | * |  | * | 8 |
| Hiller et al. 1998 |  | * | * | * | * | * | * | * | * | 8 |
| Howard et al. 2014 | * | * |  | * | * | * | * | * | * | 8 |
| Karppi et al. 2011 |  | * | * | * | * | * | * |  | * | 7 |
| Klein et al. 2003 | * | * |  | * | * | * | * | * | * | 8 |
| Kuang et al. 2013 |  | * | * | * |  |  | * |  | * | 5 |
| Leske et al. 2002 |  | * | * | * |  |  | * |  | * | 5 |
| Mares et al. 2010 |  | * | * | * | * | * | * |  | * | 7 |
| Richter et al. 2012 |  | * | * | * |  |  | * |  | * | 5 |
| Schaumberg et al. 2000 | * | * |  | * | * | * |  | * | * | 7 |
| Tan et al. 2008 |  | * | * | * | * | * | * | * | * | 8 |
| Weintraub et al. 2002 | * | * |  | * | * | * |  | * | * | 7 |
| Williams et al. 2009 | * | * |  | * | * | * |  | * | * | 7 |
| Yoshida et al. 2010 | * | * |  | * | * | * |  |  | * | 6 |

Abbreviations: BMI: body mass index, ARC: age-related cataract

^1^Quality assessment was done based on the Newcastle-Ottawa scale (NOS)
