# Supplementary figures and images for: Association between obesity and age-related cataract: an updated systematic review and dose–response meta-analysis of prospective cohort studies
Source: Front Nutr. 2024 Jan 31;10:1215212. doi: 10.3389/fnut.2023.1215212 (PMC10866009; doi:10.3389/fnut.2023.1215212)

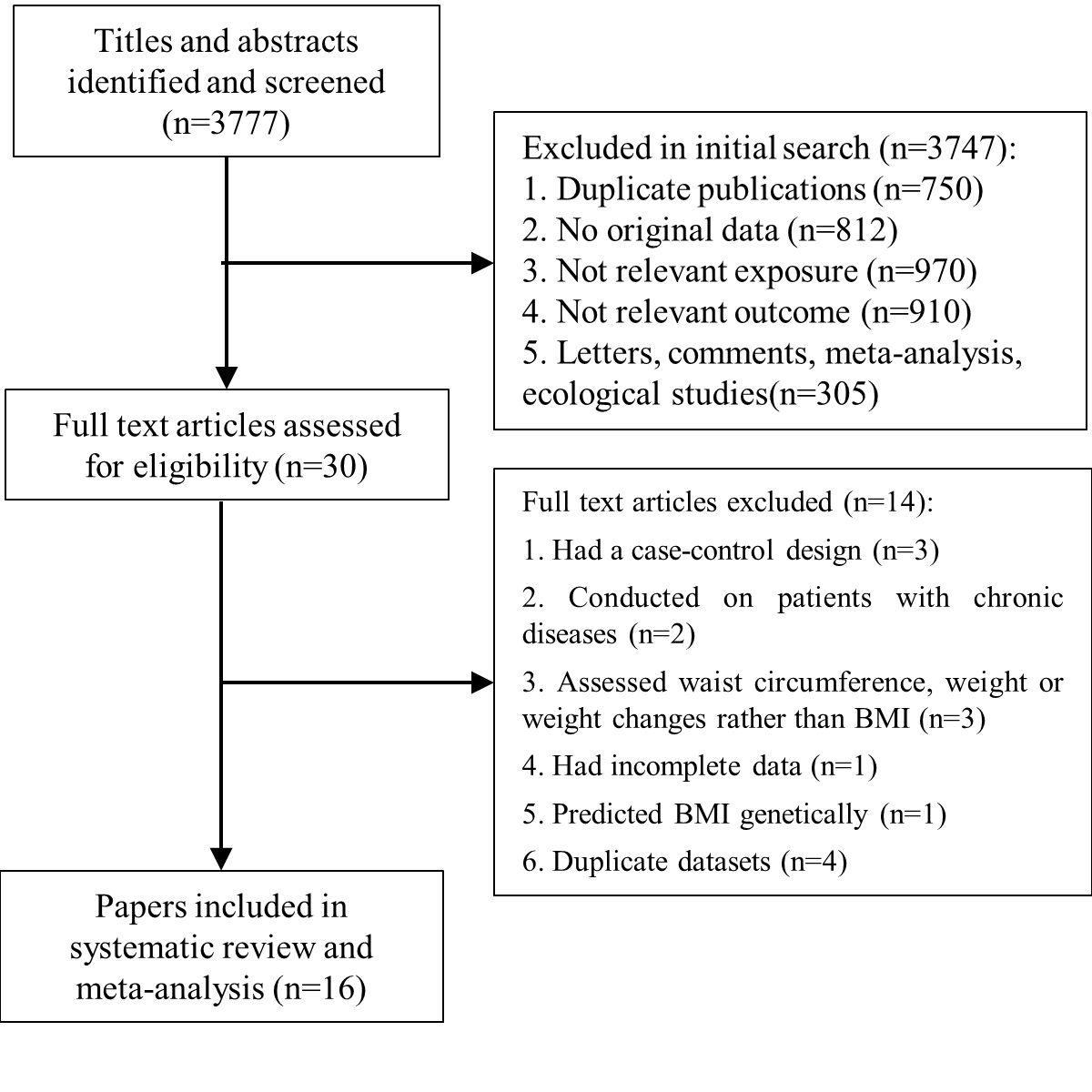

Supplement: Supplementary file 2 [file Image_1.TIF]
